# Comparison of the monocular Humphrey visual field and the binocular Humphrey esterman visual field test for driver licensing in glaucoma subjects in Sweden

**DOI:** 10.1186/1471-2415-12-35

**Published:** 2012-08-02

**Authors:** Marcelo Ayala

**Affiliations:** 1 Glaucoma Department, St. Erik Eye Hospital, Karolinska Institute, Stockholm, Sweden

**Keywords:** Visual fields, Glaucoma, Driving fitness

## Abstract

**Background:**

The purpose of this study was to compare the monocular Humphrey Visual Field (HVF) with the binocular Humphrey Esterman Visual Field (HEVF) for determining whether subjects suffering from glaucoma fulfilled the new medical requirements for possession of a Swedish driver’s license.

**Methods:**

HVF SITA Fast 24–2 full threshold (monocularly) and HEVF (binocularly) were performed consecutively on the same day on 40 subjects with glaucomatous damage of varying degrees in both eyes. Assessment of results was constituted as either “pass” or “fail”, according to the new medical requirements put into effect September 1, 2010 by the Swedish Transport Agency.

**Results:**

Forty subjects were recruited and participated in the study. Sixteen subjects passed both tests, and sixteen subjects failed both tests. Eight subjects passed the HEFV but failed the HVF. There was a significant difference between HEVF and HVF (χ^2^, p = 0.004). There were no subjects who passed the HVF, but failed the HEVF.

**Conclusions:**

The monocular visual field test (HVF) gave more specific information about the location and depth of the defects, and therefore is the overwhelming method of choice for use in diagnostics. The binocular visual field test (HEVF) seems not be as efficient as the HVF in finding visual field defects in glaucoma subjects, and is therefore doubtful in evaluating visual capabilities in traffic situations.

## Background

There is a long list of eye diseases and conditions affecting the field of vision, among them glaucoma. Glaucoma is defined as ”a disease causing damage to the optic nerve with resulting visual field defects, characterized by slow progression” [1]. Glaucoma causes damage to the optic disc, which leads to visual field defects.

The visual field is of great importance while driving; a limited field of vision hinders the driver’s capability of not only detecting objects in the periphery, but also judging distances and speed. Studies have shown that drivers with limited fields of vision have significantly poorer driving capabilities with regard to speed adjustment with lane changes, maintaining lane positions in a curve, as well as anticipatory skills [2].

Until now, there has not been a specific testing method required by the Swedish Transport Agency, and the requirement that was specified was stated simply that the applicant’s binocular visual field must be at least equivalent to a normal visual field of one eye [3].

The Humphrey Field Analyzer (HFA) is an automated, static threshold perimeter, using stimuli of varying luminance in order to find the minimum luminance which can be detected in each test point. The HFA offers several different testing programs, each with specialized testing strategies, including threshold programs which are appropriate for drivers’ license testing. Esterman visual field perimetry is a binocular testing method which is also available on the HFA. The test consists of 120 white test points shown with equal, non-adjustable suprathreshold light intensity of 10 dB and examines more than 130° of the field. This binocular method is useful in glaucoma patients with later stage bilateral visual field defects, and is used to assess the remaining visual ability or disability. An advantage of this method is that it allows for naturally-occurring binocular enhancement, in which two seeing eyes compensate for defects in the fellow eyes. During binocular viewing, each location in the right monocular field has a corresponding point in the left monocular field and vice versa. Disadvantages of this technique are that it is not possible to judge whether the defect is absolute or relative, and there is no way to control fixation stability since the binocular testing conditions eliminate naturally occurring blind spots which are used for fixation control in other tests.

Beginning September 1, 2010, new regulations for medical requirements for drivers’ licenses in Sweden went into effect [4]. The new rules outlined clear for not only which visual field testing method was to be used, but also how the results should be interpreted and assessed, and guidelines for doctors to report drivers not fulfilling the requirements. According to the rules of the Swedish Transport Agency is the ophthalmologist who decides whether to use the binocular or the monocular test in testing driving capacity. Unfortunately, these new regulations have not stated a level for reliability of the visual fields performed.

The purpose of this study was the comparison of the monocular Humphrey Visual Field (HVF) and the binocular Humphrey Esterman Visual Field (HEVF) for detection of visual field defects in subjects with glaucoma for then determining whether they fulfilled the new medical requirements for possession of a Swedish driver’s license.

## Methods

Subjects with consistent clinical diagnosis of primary open angle glaucoma (POAG) in both eyes were recruited prospectively from the Glaucoma Department at the St. Erik Eye Hospital in Stockholm, Sweden. All subjects had previously presented with glaucomatous visual field loss in both eyes confirmed using the glaucoma hemifield test (GHT). All subjects have performed at least three previous field tests before including in the study. In all cases GHT was outside normal limits. In addition, all subjects had optic disc appearance in both eyes consistent with a clinical diagnosis of POAG. Furthermore, all included subjects were tested with the Heidelberg Retinal Tomograph (HRT) version 3 (Heidelberg Engineering Inc, Heidelberg, Germany) and were classified as glaucoma according to the Moorfields Regression Analysis. All included subjects have a visual acuity better than 6/12, refraction <5 diopter ametropia, no previous ocular surgery except cataract extraction and no other posterior segment eye disease. Regarding intraocular pressure (IOP), all included subjects were on medical treatment and the IOP was below 21 mmHg at the time of inclusion. To reduce sources of error, all subjects were examined by one ophthalmologist (MA) meanwhile visual filed tests were performed by the same assistant nurse. Test sequence was randomized with time allowed for rest between tests. Subjects were also given oral instructions regarding the test, and shown a demonstration to be sure they understood how to respond to the stimuli.

Visual field testing was carried out monocularly using the Humphrey Field Analyzer (Humphrey Instruments, Dublin, CA, USA) SITA Fast 24–2 strategy (hereafter referred to as HVF) as well as binocularly using Humphrey Field Analyzer with Esterman strategy (hereafter referred to as HEVF) in a random order on the same day. Test reliability was assessed with the help of Humphrey criteria such as false positive (> 15%) and fixation errors (> 25%). Subjects whose visual field tests were deemed unreliable according to these criteria were not included in the study. Mean Deviation (MD) and Pattern Standard Deviation (PSD) were used to aid analysis of the results. Assessment of results was constituted as either “pass” or “fail”, according to current Swedish Transport Agency requirements outlined below.

In the case of binocular vision (HEFV), the applicant must have no missed points in the:

Horizontal field of vision of at least 120° in which at least 50° to the right and left of the centre of the visual field.

Vertical field of vision of at least 20° above and below the centre of the visual field.

Two adjacent missed points inside the horizontal region described above and inside of the vertical 20^o^ above and below centre constitute a “fail” of the Esterman screening for a driver’s license, and thus a barrier for possession.

In the case of monocular vision (HVF) the threshold values, e.g. the weakest stimulus which elicited a response at each test point, must be:

At least 20 dB within a radius of 10° from the centre of the visual field

At least 10 dB within a radius of 20° from the centre of the visual field

The study complied with the tenets of the Declaration of Helsinki and was approved by our institutional human experimentation committee, with all subjects giving informed consent before participation.

### Statistical analysis

Correlations between the HEVF and HVF were calculated with Chi-Square (χ^2)^) Test. The level of statistical significance was set at 0.05.

The statistical analysis was performed using the STATA software (Statacorp,4905 Lakeway Drive, College Station, Texas 77845, USA).

## Results

Forty subjects with diagnosed glaucoma participated in the study. The average age of the subjects was 70 years old (range 56–82 years). Gender distribution: males/females: 22/18. The sample mean Humphrey MD (mean deviation) was −11.6 dB (SD 4 dB; range −4.48 to −18.82 dB) for the right eye and −11.7 dB (SD 4.4 dB; range −5.36 to −19.04 dB) for the left eye. Table 1 shows the results of the examinations. Results showed that twenty four (60%) patients received a ”pass” score with HEVF, while sixteen (40%) subjects passed the HVF. All subjects who passed the HVF also passed the HEVF, but eight subjects who received a “fail” score on the HVF passed the HEVF anyway. These eight subjects that failed according to HVF but passed according to the HEVF showed moderate glaucoma damage in the visual fields with an average MD = −9.19 (SD = 3.44). No subject who failed the HVF but passed the HEVF showed advanced glaucoma. Subjects who passed the test using HVF showed an average MD = −7.79 (SD = 3.24) meanwhile subjects who failed both tests showed an average MD = −13.30 (SD = 5.23). In all, sixteen subjects passed both tests; sixteen subjects failed both tests (see Table 1).

**Table 1:** Comparison of the monocular HFV with the binocular HEFV tests

		**Binocular**	**HEVF**
		**Fail**	**Pass**
Monocular	Fail	16	8
HVF	Pass	0	16

There was a significant difference between HEVF and HVF (χ^2^, p = 0.004) when testing for “pass” or “failed”.

## Discussion

Results indicate that there was a significant difference between HEVF and HVF. The findings of the study demonstrated that more subjects passed the new driver’s license visual field requirement using the HEVF when compared to the HVF.

Results indicate that the HVF is still the predominant visual field test for the detection and diagnosis of visual field defects in glaucoma, but in the case of driver’s license screenings, the HEVF can be as effective as the HVF in detection of central defects in cases of advanced glaucoma.

Crabb et al in 1998 described a method known as integrated visual field (IVF) to simulate a binocular visual field using data from monocular visual fields [5]. The IVF is estimated from monocular results, taking the best sensitivity values from corresponding visual fields locations from the two eyes. The authors found a substantial agreement between the simulated binocular results and HEVF in classifying glaucomatous patients [5]. These results were even corroborated by Nelsson-Quigg et al., which demonstrated general agreement between results from the HEVF and an integration of two monocular visual fields into one binocular field [6].

Since the current study did not use IVF data compilation it was not possible to make direct comparisons to previous research. Further studies must be done to determine agreement between the IVF and the HEVF in classifying a glaucomatous subject’s legal fitness to drive according to the guidelines of the Swedish Transport Agency. Probably the HEVF can be replaced by the IVF improving visual field evaluation and saving resources.

**Figure 1:**
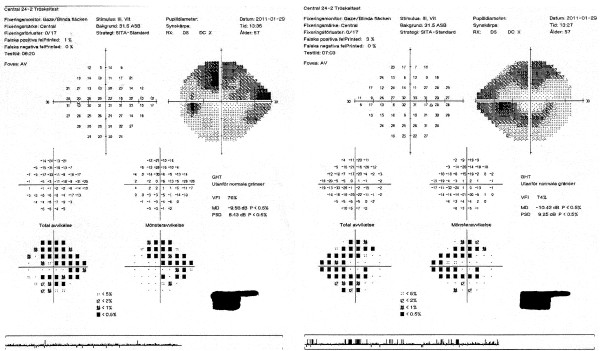
**HVF of one subject’s right and left eyes.** Even though the bilateral field defects resulted in a “fail” result for the HVF, the subject passed the HEVF (see Figure 2).

**Figure 2:**
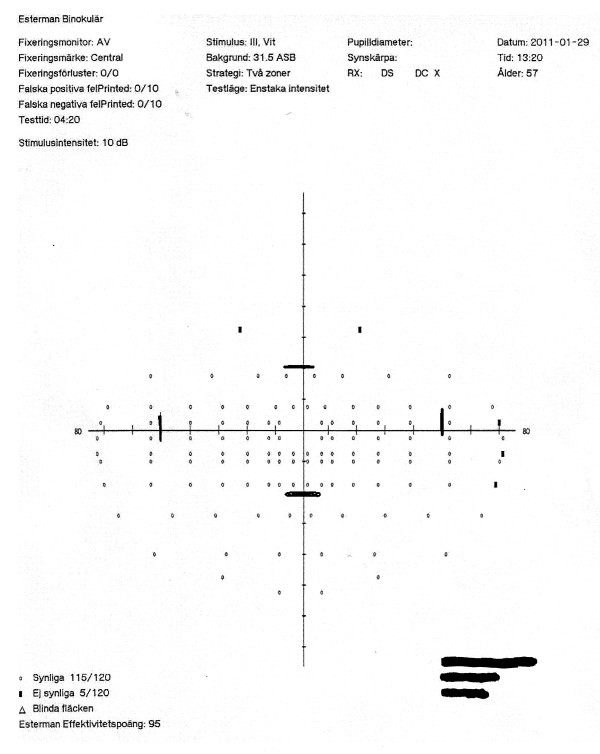
**The same subject’s HEVF shows only a few missed points.** The remaining defect is not in the vertical area 20° from the center of the visual field (denoted by thick black tick marks), and the missed adjacent points are outside of the horizontal area 50° from the center (also denoted by thick black tick marks), so it is still classified as “pass”.

**Figure 3:**
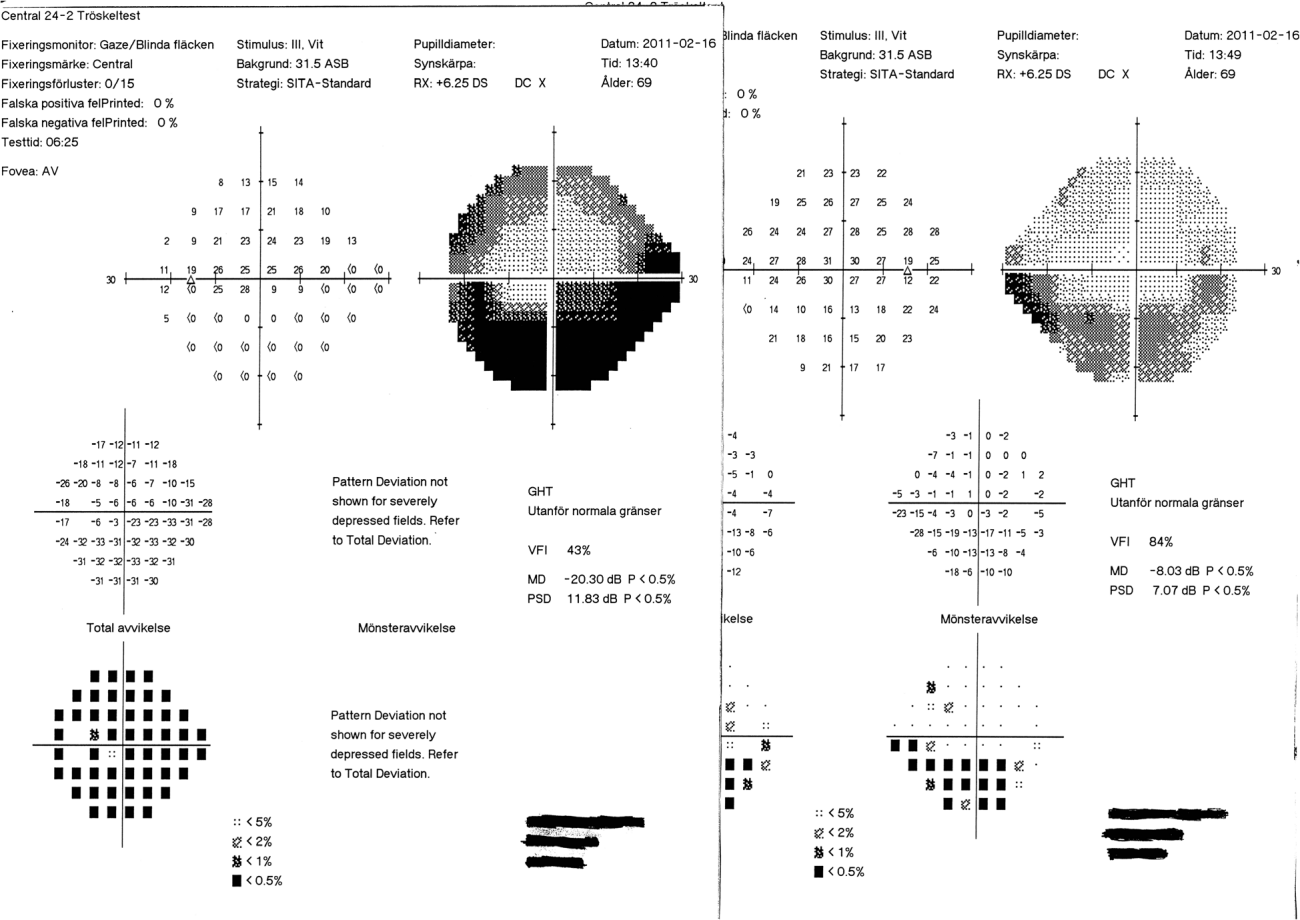
**An HVF test from one of the subjects included.** Missed test points can be seen with sensitivity values below 10 dB within the area of defect; there are many missed adjacent points in the required field. The test is therefore classified as “fail”.

**Figure 4:**
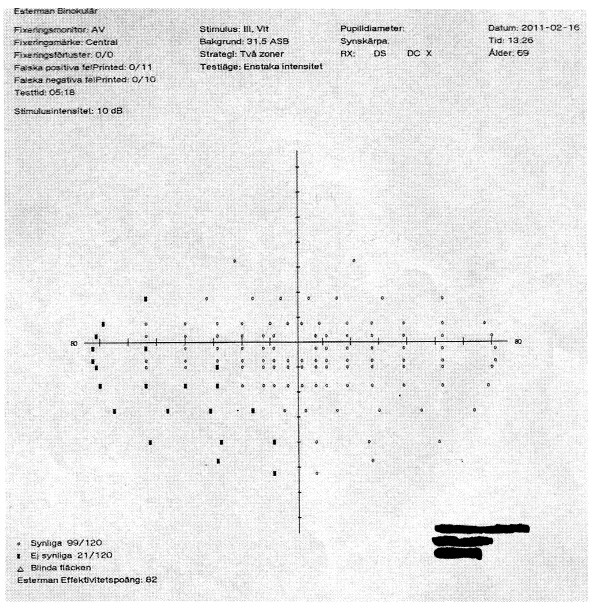
**The same subject’s HEVF shows lot of missed points.** The defects (denoted by thick black tick marks) are situated in the vertical area 20° and the horizontal 50° from the center. In this area 3 adjacent defects were detected, therefore the test is classified as “fail”.

According to the new regulations, consideration is given not only to the central visual field, but also the peripheral portions of the visual field. In previous studies, testing was focused only on the central 20° of the visual field. A higher ”pass” rate for the HEVF could be due to the fact that even though most of the subjects had peripheral visual field defects, the defects were not large or deep enough to constitute a ”fail” result (see Figures 1 and 2) under the new binocular testing regulations. The Figures 3 and 4 belong to the same subject who failed both the HFV and the HEVF.

These new Swedish regulations are intended to be closure to regulations in other European countries. In the UK a driver should have a binocular horizontal visual field of at least 120° assessed using a Goldmann III4e target or similar and have no significant defect [7]. The European Union Member States have their individual driver’s license requirements and guidelines, but the European Union regulations can potentially overrule these. According to the European Union Commission Directive put into effect August 25, 2009 (amending a directive from 1991), new visual requirements were recommended in the European Union for obtaining a driver’s license. Applicants shall have a visual acuity of at least 0.5 when using both eyes together. Moreover, the horizontal visual field should be at least 120 degrees; the extension should be at least 50 degrees left and right and 20 degrees up and down [8]. However, no description of which visual field test to be used has been included in the European Commission’s regulations.

The new Swedish regulations for fitness to drive have no stated any influence in fitness to drive and where the visual field defects are localized. It does not matter if visual defects are placed in the nasally or temporally part. Theoretically temporal defects would alter more driving capabilities than nasal defects. In case of nasal visual field defects, the visual fields from the other eye will compensate. Furthermore, vehicles coming from the sides will be detected mostly with the temporal part of the visual field. Information about influence of placement of visual field defects and fitness to drive are scarce in the literature. Racette & Casson found no difference in driving capabilities tested on-road driving with different locations of visual field defects when testing subjects that were affected of cerebral vascular accident [9]. The authors even concluded that the results must be reconfirmed because of a large individual difference and small sample size. Driving performance is difficult to measured as it was pointed out by Crabb et al , because of accident rates are low in the general population, driving simulators are difficult to utilise and other factors than visual defects can alter driving fitness in glaucoma subjects like age and cognitive skills [10].

According to the Swedish Transport Agency is up to the ophthalmologist to decide to use whether the monocular or the binocular visual field tests for determining fitness to drive. The decision should be based on clinical findings. Subjects included in the present study showed moderate visual field impairment due to glaucoma (MD = −11 dB). Haymes et al evaluating glaucoma subjects with slight glaucoma damage (MD = −6.5 dB) found no increased difficulties while driving using a real-world setting compared to normal subjects [11]. It is very possible that subjects affected by slight glaucoma damage would passed both tests as same as subjects with great visual field damage would failed in both tests. Further investigations should be done to correlate results from monocular and binocular tests methods with “real life” situations during driving. Studies using “driving simulators” would add more valuable information.

## Conclusions

The findings of this study clearly show that the HEVF is an easy-to-use method for both patient and examiner, which probably gives a more realistic picture of the patient driver’s visual field. However, the HEVF is a suprathreshold test in which fixation can not be assured. Subjects with borderline ”pass” results from an HEVF should be retested with a HVF for more precise mapping of any defects. Further studies should be performed with a larger group of subjects to investigate more closely the usefulness and accuracy of the Esterman method in driver’s license vision screening.

## Competing interests

The author declares that he has no competing interests.

## Author’s contributions

MA carried out the whole study.

## Pre-publication history

The pre-publication history for this paper can be accessed here:

http://www.biomedcentral.com/1471-2415/12/35/prepub
